# Supplementary material for: Prenatal Ultrasound Findings and Chromosomal Outcomes of Pregnancies with Mosaic Embryo Transfer
Source: Diagnostics (Basel). 2024 Dec 12;14(24):2795. doi: 10.3390/diagnostics14242795 (PMC11674424; doi:10.3390/diagnostics14242795)
Supplement: Supplementary file 1 [file diagnostics-14-02795-s001.zip › diagnostics-3346495-supplementary.pdf]

Supplementary Figure S1.

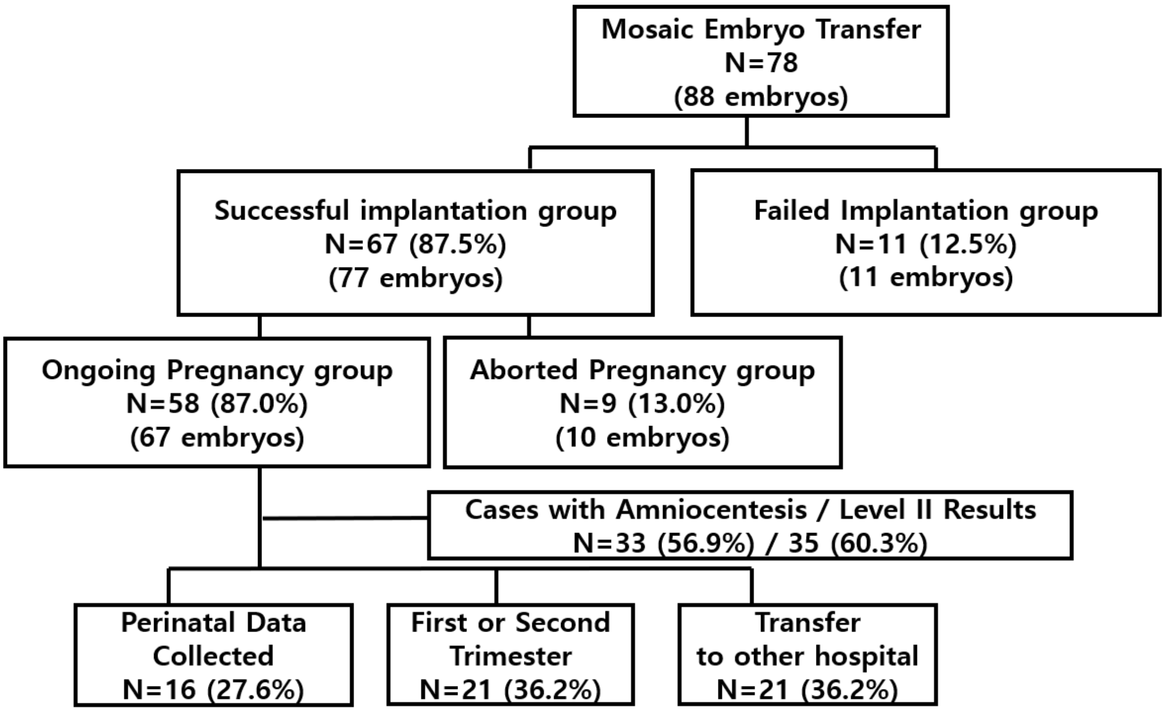


This figure shows the flow chart of the study group. The number (N) refers to the number of mothers: the total study group included 78 mothers, the successful implantation group included 67 mothers, and the ongoing pregnancy group included 58 mothers. At the bottom of the figure, the number of embryos for each group is specified. In the Ongoing Pregnancy group, while 67 embryos were transferred among 58 patients, all resulted in singleton pregnancies with one embryo implanting per patient. Amniocentesis was performed for 33 mothers in the ongoing pregnancy group. Excluding those who were either still pregnant or transferred to another hospital, a total of 16 mothers were able to provide perinatal data, as they gave birth at our institution.
